# Indents

**Published:** 1912-06-15

**Authors:** 


### INDENTS.

**☞Brief Articles of Technical or Scientific Value will receive notice and comment. Contributions invited.**

*Method of Disinfecting a Tooth with Dead Pulp.* An easy method of disinfecting a tooth with a dead pulp and to reduce severe pain in inflammation due to acute peri-odontitis, when the pulp chamber and canals are not filled, consists of the following: The cavity is washed out and dried. A pledget of cotton is saturated with tincture of iodin, and to it a large ball burnisher made quite red hot is applied. This will cause the vapor of the iodin to permeate the root-canals, and if applied three times a day should reduce the acute pain and also stay the inflammatory process, if this have not advanced too far.

The same method can be used to disinfect roots before opening, adding a very small quantity of formaldehyde solution to the iodin.—E. Dunn, *Garretsonian.*

*Stick Solder.* Stick solder is made by cutting the solder in strips, but not quite to the end of the piece, making the cut alternately from each end, so that it may be bent to form one long strip. For delicate soldering operations such as are encountered in crown and bridge work, this form is preferred by some workmen. It is used with a soldering fluid composed of a saturate solution of borax to which is added vary little, a few drops only, of hydrochloric acid. The point where the solder is desired is heated to about the fusing point of the solder, the end of the strip is dipped into the soldering fluid and then pressed where it is needed. It is more under control than is solder cut into small squares. It carries with it to the point where it is needed enough, and not too much, flux. The flux is always clean. There is no delay in adding more solder as is the case when squares are used, and there is less risk of getting too much.—*Dental Brief.*

*Liquid Celluloid,* made by dissolving celluloid in acetone, should be kept on hand in the laboratory at all times for repairing plaster models and cut or scratched hands. No heat is required.—V. C. Smedley in *Digest.*

*Just Fees.* A just fee can be determined only by considering the average amount of results that should be accomplished with average industry, the average amount of "work" one has the opportunity to procure to do, the average length of time a dentist should expect to be able to practise, the necessities of the average dentist and the average practice and the conditional rights of rest, recreation and mental and social improvement that industry, sobriety and success usually confer.—*Digest.*

*Cleaning Nerve Broaches.* Nerve broaches, especially when clogged with putrid pulp debris, are readily cleaned, if the broaches are pushed through a tightly spread piece of rubber-dam. The breaking of a broach during this procedure is an indication that its point was dull or that it was intrinsically weak. At any rate, it is better to break a broach in this way than to have it break in the root canal.—R. Sursen.

*Some "Don'ts" in Pulp Anesthetization.* 1. "Don't" remove pulp by anesthetization without first applying the rubber dam, for "isolation's" sake.

2. "Don't" use anesthetizing agent without a thorough removal of all caries, for "absorption's" sake.

3. "Don't" use anesthetizing agent until cavity is thoroughly disinfected, for "asepsis'" sake.

4. "Don't" prolong pressure until the surrounding tissue is so infiltrated with the solution that recovery is not prompt, for "pericementitis'" sake.

5. "Don't" enlarge pulp chamber or canal opening with non-sterile bur, for "cleanliness'" sake.

6. "Don't" use broach that has not been previously sterilized or disinfected, for "Hec's" sake.

7. "Don't" use broach without testing, for the "tooth's" sake.

8. "Don't" wrap cotton on broach with bacteria laden fingers, if only for "goodness'" sake.

9. "Don't" use "peroxide" to check hemorrhage or cleanse canal, for "color's" sake.

10. "Don't" fill immediately, but seal in an anodyne agent and fill at a subsequent sitting for "thoroughness'" sake.—Dr. Weber in *Summary*.

*Controlling Hemorrhages in Setting Crowns and Bridges.*

In irritated conditions, where the gums have a tendency to weep and bleed, treat the gum margin with 15 per cent. solution of Trichloracetic acid. I know of no other astringent that so absolutely controls such a condition in setting crowns and bridges and inlays, and at the same time having such a curative effect. —J. E. Argue, *Dental Review*.

*Suggestions on Nitrous Oxide Anesthesia.*

Snoring after the administration of nitrous oxide for a short period of time and laryngeal stertor must be carefully discriminated. Patients with thick pendulous uvulae begin to snore a few seconds after inhalation has commenced. This is also true of patients having post-nasal adenoids and enlarged tonsils; also those having loose baggy cheeks. To presume such snoring a sign of anesthesia and to operate with no further testimony to the fact that anesthesia has ensued would be committing an egregious blunder. Snoring *per se* is not stertor. True stertor is caused by the vibration of the aryteno-epiglottidean folds, and is more musical. A group of symptoms that anesthesia has ensued would, to my mind, be a far safer guide to the anesthetist, and would be more scientific. Patients will snore and not be anesthetized. I usually watch breathing very carefully, but if the kind of breathing would indicate anesthesia while the conjunctival reflex persists, the return to consciousness will be very rapid. Note the following changes: Duskiness of skin; ears and finger-tips darker; pupils dilate; eyes dull and expressionless; squinting, and if pure nitrous oxid be administered to children, minus oxygen, and early jactitation occurs, which will often preclude the possibility of an operation. Quivering of the orbicularis palpebrarum warns us that jactitation is about to commence. Oxygen administered with nitrous oxide at this time is advantageous, because the anesthetic qualities of the nitrous oxid progress and the asphyxial symptoms are abolished.—C. F. B. Stowell, D.D.S., Chicago, Ills.

*A Valuable Root Filling.* A preparation of gutta-percha that was suggested by Dr. R. C. Cochran, some years ago, led me to some of the many experiments I made along this line. It opened up the possibilities of a new root canal filling that seemed to suggest to me many important features. Undoubtedly, you are all familiar with the preparation of this agent, but I want to suggest it again for a little further consideration. Dr. Cochran's method is simply as follows: Dissolve gutta-percha in chloroform and when the excess of chloroform has evaporated add eucalyptol, and as much thymol is added to this as the oil will take up. Then grind this compound up to a consistency that can easily be handled, leaving it in this state until it is to be used. But in these experiments I used the eucalyptol without the thymol. If the canal has been sterilized the thymol is too irritating and is unnecessary. Now what really happens in this preparation is simply this: A certain portion of the chlorin remains in the gutta-percha and the essential oil helps to retain that substance in a combination with the gutta-percha and renders it into a workable state. The chlorin that remains in the gutta-percha and the terpine that is in the essential oil combine and form a dihydrobenzol, which has very efficient antiseptic properties. Dihydrobenzol also has some irritating properties due to an oxidizing process that goes on for some little time, especially if there is much organic moisture in the root canal. I don't wish to be understood that this substance has a complete destructive power on bacteria, and especially on the spores of bacteria, but that it has a strong inhibitory action, and with some bacteria a germicidal power. The staphylococcus and the typhoid bacillus were destroyed in root canals by this agent, but the anthrax bacillus would after ten or twelve days develop in the culture media.—Geo. B. Cook in *Review*.

*Keep the Tooth-Brush Clean.* Another time-honored and long-cherished institution has been attacked by the ruthless scientist and hygienist. This time it is the tooth-brush. Soap and the tooth-brush have hitherto been considered almost indispensable adjuncts of civilization. After years and years of patient teaching of the young that correct personal hygiene required the use of the tooth-brush, after its adoption as a necessary or desirable part of the accouterment of the United States soldier, who proudly carried it, stuck in the band of his hat, in the campaigns in Cuba, Porto Rico, the Philippines, and in the Boxer rebellion in China, where it was observed and commented on favorably by the observers of the sanitary departments of the armies of the world, it is now to be discredited because it is not conformable to modern hygiene and sanitation. The tooth-brush, according to Smale and Jones becomes septic after being used once, each bristle becoming an inoculating needle by which the person using it may become inoculated with the multitudinous germs which flourish on it, producing pyorrhea alveolaris, with such serious possible sequelae as anemia, gastritis and arthritis. They propose as a solution of the difficulty that a new brush be used each time, or that the brush be boiled for five minutes after each using, or that it be kept in a one per cent. solution of tricresol or a ten per cent. solution of formaldehyde. It is a bit disconcerting to think that we have been going along all these years teaching the correctness of the use of the tooth-brush, only to find that instead of being an aid to good personal hygiene, it has been an instrument for creating or perpetuating infections. How long will it be before that remaining bulwark of civilization —soap—is also attacked, and the warning given that the unctuous substance that we smear over our faces and bodies daily is nothing more nor less than an emulsion of deadly bacteria, and that it is only because of our high degree of natural resistance that we have not succumbed? Seriously, however, the contention of Smale and Jones should not be ridiculed, nor, on the other hand, should this useful instrument be discarded, lest a worse state follow. But the danger of auto-inoculation with bacteria from the mouth by the brush is perhaps not to be feared so much as its contamination by extraneous matter from lack of proper care in keeping. The methods of disinfection suggested by Smale and Jones, while ideal, are not practical, because they will not be carried out. An effective method, and better because practical and simple, would be to wash the brush thoroughly after using, rinse it in a antiseptic solution—boric acid, for instance—and then keep it in a bottle corked with antiseptic cotton. The hygienic condition of the tooth-brush is certainly a matter worthy of attention.—*Journal A. M. A.*

*Preparing Sensitive Teeth for Gold Crowns.* If, when grinding teeth preparatory to inserting gold crowns, three or four thin carborundum separating disks are mounted together on the screw mandrel, instead of the heavy stones ordinarily used, it will be found that by moving the disks back and forth over the surface of the tooth it is possible to cut much more quickly, and there will be much less discomfort to the patient, as there is less friction. The spaces between the disks hold the water far better than the surface of the solid stone.—J. Neales, D.D.S., Providence, R. I., in *Dental Cosmos.*

